# Development and validation of a dementia screening tool for primary care in Taiwan: Brain Health Test

**DOI:** 10.1371/journal.pone.0196214

**Published:** 2018-04-25

**Authors:** Ping-Huang Tsai, Jian-Liang Liu, Ker-Neng Lin, Chiung-Chih Chang, Ming-Chyi Pai, Wen-Fu Wang, Jen-Ping Huang, Tzung-Jeng Hwang, Pei-Ning Wang

**Affiliations:** 1 Division of Neurology, Department of Internal Medicine, National Yang-Ming University Hospital, Yilan, Taiwan; 2 Center for Dementia Care and Evolution, National Yang-Ming University Hospital, Yilan, Taiwan; 3 Department of Neurology, School of Medicine, National Yang-Ming University, Taipei, Taiwan; 4 Division of Neurology, Department of Medicine, Taipei City Hospital Heping Fuyou Branch, Taipei, Taiwan; 5 Department of Neurology, Neurological Institute, Taipei Veterans General Hospital, Taipei, Taiwan; 6 Department of Psychology, Soochow University, Taipei, Taiwan; 7 Department of Neurology, Kaohsiung Chang Gung Memorial Hospital, Kaohsiung, Taiwan; 8 Division of Behavioral Neurology, Department of Neurology, National Cheng Kung University Hospital, College of Medicine, National Cheng Kung University, Tainan City, Taiwan; 9 Alzheimer's Disease Research Center, National Cheng Kung University Hospital, College of Medicine, National Cheng Kung University, Tainan City, Taiwan; 10 Department of Neurology, Changhua Christian Hospital, Changhua, Taiwan; 11 Department of Psychiatry, Taipei Veterans General Hospital, Taipei, Taiwan; 12 Department of Psychiatry, College of Medicine and National Taiwan University Hospital, National Taiwan University, Taipei, Taiwan; 13 Brain Research Center, National Yang-Ming University, Taipei, Taiwan; 14 Aging and Health Research Center, National Yang Ming University, Taipei, Taiwan; Banner Alzheimer's Institute, UNITED STATES

## Abstract

**Objectives:**

To develop a simple dementia screening tool to assist primary care physicians in identifying patients with cognitive impairment among subjects with memory complaints or at a high risk for dementia.

**Design:**

The Brain Health Test (BHT) was developed by several experienced neurologists, psychiatrists, and clinical psychologists in the Taiwan Dementia Society. Validation of the BHT was conducted in the memory clinics of various levels of hospitals in Taiwan.

**Participants:**

All dementia patients at the memory clinics who met the inclusion criteria of age greater or equal to 50 years were enrolled. Besides the BHT, the Mini-Mental State Examination and Clinical Dementia Rating were used to evaluate the cognition state of the patients and the severity of dementia.

**Results:**

The BHT includes two parts: a risk evaluation and a cognitive test (BHT-cog). Self or informants reports of memory decline or needing help from others to manage money or medications were significantly associated with cognitive impairment. Among the risk factors evaluated in the BHT, a total risk score greater or equal to 8 was defined as a high risk for dementia. The total score for the finalized BHT-cog was 16. When the cutoff value for the BHT-cog was set to 10 for differentiating dementia and a normal mental state, the sensitivity was 91.5%, the specificity was 87.3%, the positive predictive value was 94.8%, and the negative predictive value was 80.1% The area under the receiver operating characteristic curve between dementia and healthy subjects was 0.958 (95% CI = 0.941–0.975).

**Conclusions:**

The BHT is a simple tool that may be useful in primary care settings to identify high-risk patients to target for cognitive screening.

## Introduction

Dementia refers to a broad category of brain diseases that present as a progressive decline in cognitive function beyond what is expected from normal aging. The size of the dementia population is projected to increase, with substantial societal impacts on health care costs and caregiving. It was estimated that 35.6 million people lived with dementia worldwide in 2010, with numbers projected to nearly double every 20 years, to 65.7 million in 2030 and 115.4 million in 2050[1]. Although the U.S. Preventive Services Task Force does not provide a recommendation for or against screening for cognitive impairment[2], many dementia patients are unaware of their impaired cognitive function until they are screened. According to our community-based “The Yilan Study”[3] in Taiwan, 76% of suspected dementia patients do not receive a definite diagnosis at the hospital. A dementia screening accompanied by routine health checks by assistant of primary care physicians can accelerate the dementia diagnosis.

Not only in the community, dementia is also highly unrecognized in medical settings, where between 27% and 81% of affected individuals are inadequately diagnosed[4–6]. Primary care physicians fail to make a diagnosis for up to 66.7% of patients with dementia, especially those with mild dementia[4]. Numerous factors affect the recognition of cognitive impairment, and previous studies have reported that objective tests can identify impaired subjects more accurately than physicians in regular clinical encounters[7]. Accordingly, recent guidelines suggest that the use of a brief, structured cognitive assessment tool correctly classifies patients with dementia more often than detection by the patients' primary care physicians [6].

The Mini-Mental State Examination (MMSE) [8] is widely used for the screening of global cognitive function. However, the reliability of cognitive abilities tests, such as MMSE, may be compromised by numerous factors, such as age, the degree of education [9–14], and the proficiency of the research assistants administering the test [15]. Additionally, no comprehensive screening tool measures other risk factors for dementia such as comorbidities, head trauma, and depression. As such, there is a need for the development of a screening tool that incorporates potential risks to screening possible dementia patients by primary care physicians.

In Taiwan, the Health Promotion Administration (HPA), Ministry of Health and Welfare (MOHW) provides the basic health examination once every 3 years for individuals older than 45 years old and annually for those older than 65 years old. This annual basic health check-up is mostly performed by the primary care or family physician. Therefore, our goal was to develop an easy-to-use clinical tool, which uses information already available or easily obtainable during basic health examination, to identify likely dementia patients based on risk factors, and to perform a targeting cognitive screening test.

## Materials and methods

### Assessment tasks (Brain Health Test)

Several experienced neurologists, psychiatrists, and clinical psychologists in the Taiwan Dementia Society assembled a task force in August 2014 to develop a dementia screening test called the Brain Health Test (BHT). After three consensus meetings and two workshops, the BHT was finalized in January 2015. All components of the BHT were selected based on the principles of early detection, proper feasibility, and short administration time.

The BHT is composed of two parts: 1) risk factors, including age, education level, subjective memory complaints (subjects, informants or medical staff), body mass index (BMI), history of stroke, diabetes, hypertension, hyperlipidemia, head trauma accompanied by consciousness change, needing assistance to manage money or medications, and depression, defined as depressed mood or a loss of interest or pleasure in daily activities for more than two weeks; 2) a brief cognitive test (BHT-cog), including orientation to time, immediate and delayed recall of five items, categorical verbal fluency test (listing four-legged animals in one minute), and the Clock Drawing Test (CDT) (10:10). The original BHT, English version, is attached as a supplement figure (S1 Fig).

The risk factors were captured from the previous studies, in which numerous studies were based on Taiwan's National Health Insurance Research Database [16–27], and were simplified not to increase clinical loading according to the basic health examination, that was provided by HPA, MOHW.

### Study design

This was a multi-site study, including Taipei Veterans General Hospital, Kaohsiung Chang Gung Memorial Hospital, National Cheng Kung University Hospital, Changhua Christian Hospital, National Yang Ming University Hospital, and Taipei City Hospital Heping Fuyou Branch. These sites represent different hospital levels in Taiwan, including four medical centers and two regional hospitals. Additionally, these sites are located in different areas in Taiwan, including northern, middle, southern, and northeastern Taiwan, serving urban to rural populations. Normal cognitive elders, patients with mild cognitive impairment and patients with dementia were enrolled in this study to validate the sensitivity and specificity of BHT.

According to the formula, which was provided by Dillman[28], for estimating desired sample sizes, the minimal sampling size was 385 with the background of the number of elderly in Taiwan was 2.4 million and the prevalence of all-cause dementia was 8.04%[17].

The research was approved by the institutional review board (IRB) of every hospital. Written informed consent was obtained from all participants in the study. For patients with dementia, all dementia patients and their proxies provided written, informed consent. For MCI patients, the inform consent could be signed by the patients themselves only, because IRB agreed that patients with MCI, who suffered from as a change in cognition with impairment in one or more cognitive domains but no evidence of impairment in social or occupational functioning, were able to understand the study procedures and non-invasive assessments were performed in this study. We also explained to MCI patients’ proxies about the study, if they came with the patients. The study participants were enrolled between January 2015 and April 2016.

### Assessments

All dementia patients were recruited from neurological clinics with the inclusion criteria of age greater or equal to 50 years. All participants received BHT examination before other cognitive assessments. Dementia was diagnosed according to the National Institute on Aging and Alzheimer's Association (NIA-AA) criteria [29]. After BHT, trained research assistants also administered the Chinese version of the Mini-Mental State Examination (MMSE) [8], which features a total score of 30. The Clinical Dementia Rating (CDR) was also used to determine the severity of dementia after a neurologist or a psychologist conducted separate semi-structured interviews with the patient and a knowledgeable informant. The scores for CDR are as follows: 0 for normal, 0.5 for mild cognitive function impairment (MCI), or very mild dementia (VMD)[30, 31], 1 for mild dementia, 2 for moderate dementia, and 3 for severe dementia[32]. People with VMD had mild impairment in two or more cognitive domains as well as a slight decline in daily functions, whereby the cognitive deficits were sufficient to interfere with their independence in daily life as a result of an abnormality in community affairs or at-home hobbies or as a result of personal care as assessed by the CDR. MCI was diagnosed, based on the criteria recommended by the NIA-AA, as a change in cognition with impairment in one or more cognitive domains but no evidence of impairment in social or occupational functioning as assessed by the CDR, activities of daily living (ADL)[33], and instrumental activities of daily living (IADL)[34].

After being screened for cognitive impairment, the participants received further medical, neurological, neuropsychological, and psychiatric assessments, as well as blood examinations. The neurological assessments for each of the participants included a cerebral computed tomography scan to rule out intracranial pathologies (i.e., brain tumors or stroke) that may have contributed to the cognitive decline.

The healthy control came from two resources. Most of the healthy controls were recruited from the neurological clinics as volunteers. The other healthy controls were recruited from “The Yilan Study,"[3] a community-based cohort study in Yilan City. The inclusion criteria were 1) Ascertain Dementia 8 (AD8)[35] score of zero, 2) Geriatric Depression Scale Short form (GDS-S)[36] score of zero, 3) functional status, assessed using the ADL scale[33] and IADL scale[34] as totally independent.

### Statistical analyses

Descriptive statistics were presented as means ± standard deviation. SPSS (version 22.0) for Windows (SPSS Inc., Chicago, IL, USA) was used for statistical analyses. Baseline demographic characteristics, including age, MMSE, and CDR were coded as continuous variables. Other demographic characteristics, such as sex, less than 6 years of education (<6 years), BMI (<18), history of stroke, diabetes, hypertension, hyperlipidemia, head trauma accompanied by consciousness change, needing assistance to manage money or medications, and depression were coded as dichotomous variables. The total scores for BHT-cog were coded as a continuous variable. One-way analysis of variance (ANOVA) and a multivariate general linear model were used to analyze demographic factors or interactions with BHT-cog scores. Post-hoc analysis with the Tukey method was used to avoid type one error in multiple comparisons. Pearson correlation analysis was used to check the correlations between scores for BHT-cog and in other demographic data studies. Receiver operating characteristic (ROC) was used to evaluate the discriminatory potential of BHT-cog scores for dementia screening. The cutoff scores for BHT-cog and their sensitivity and specificity were determined for the dementia and healthy control groups. All of the statistical tests were two-tailed, and significance levels were set at a p-value of less than 0.05.

## Results

Eight hundred and sixty-seven subjects were enrolled in this study initially, and 54 subjects were excluded due to incomplete data. Among the remaining 813 subjects (445 women and 368 men, mean age of 76±9 years, mean education of 7.7±4.9 years), 166 (20.4%) were healthy, 225 (27.7%) had MCI, and 422 (51.9%) had dementia. Most (75.8%, 320/422) of the dementia patients had Alzheimer’s disease (AD). Other types of dementia included 33 (7.8%) cases of vascular dementia, 7 (1.7%) cases of dementia with Lewy’s body, 9 (2.1%) cases of Parkinson’s disease with dementia, 7 (1.7%) cases of frontotemporal dementia, and 46 (10.9%) cases of mixed type dementia. Among the dementia patients, 60 had VMD (CDR = 0.5), 279 had mild dementia (CDR = 1), 66 had moderate dementia (CDR = 2), and 17 had severe dementia (CDR = 3). In the analysis, we separated these patients into three groups: healthy, MCI, and dementia subjects.

### Demographic data

Table 1 presents the demographic variables in the three groups studied: healthy, MCI, and dementia subjects. In our study, older age and lower education were more common in the dementia patients, and the difference was significant in comparison with healthy and MCI subjects.

**Table 1:** The characteristics of the three groups studied: healthy, MCI, and dementia subjects.

	Healthy (n = 166)	MCI (n = 225)	Dementia (n = 422)	*p* value
Age (years)	72.3±8.4	73.3±8.7	79.0±8.4*+	0.000
Gender (Female, n, %)	87 (52.4%)	119 (52.9%)	239 (56.6%)	0.527
Education (<6 years, n, %)	20 (12%)	36 (16%)	117 (27.7%)*+	0.000
MMSE	27.9±2.1	24.6±3.8*	16.8±6.3*+	0.000
Memory decline (subjects, n, %)	62 (37.3%)	163 (72.4%)*	286 (67.8%)*	0.000
Memory decline (informants, n, %)	39 (23.5%)	141 (62.7%)*	362 (85.8%)*+	0.000
Memory decline (subjects or informants, n, %)	72 (43.4%)	209 (92.9%)*	405 (96%)*	0.000
Memory impairment (doctors, n, %)	16 (9.6%)	24 (10.7%)	76 (18%)*+	0.006
BMI (<18, n, %)	7 (4.2%)	10 (4.4%)	18 (4.3%)	0.932
Stroke (n, %)	22 (13.3%)	37 (16.4%)	72 (17.1%)	0.522
Diabetes (n, %)	41 (24.7%)	64 (28.4%)	124 (29.4%)	0.513
Hypertension (n, %)	930 (56%)	121 (53.8%)	238 (56.4%)	0.810
Hyperlipidemia (n, %)	44 (26.5%)	61 (27.1%)	108 (25.6%)	0.912
Head trauma with consciousness change (n, %)	6 (3.6%)	13 (5.8%)	44 (10.4%)*	0.009
Needing assistance to manage money or medications (n, %)	9 (5.4%)	47 (20.9%)*	262 (62.1%)*+	0.000
Depression (n, %)	14 (8.4%)	33 (14.7%)	120 (28.4%)*+	0.000

Note: Data are presented as mean ± standard deviation. The *p*-value was obtained from one-way analysis of variance. Abbreviations: MCI: mild cognitive impairment; MMSE: Mini-Mental State Examination; BMI: Body mass index

* *p* < 0.05 compared with control

+ *p* < 0.05 comparing MCI with dementia

### Subjective memory complaints

Only 67.8% of the dementia patients self-reported memory decline, whereas 85.8% of the dementia patients’ informants reported worsening of the patient’s memory, which was significantly higher than for healthy subjects. From another perspective, the percentage of informants that reported subjective memory decline in patients was 62.7% for MCI patients and 85.8% for dementia patients, which was also significantly higher than for healthy subjects. If we combined the subjective memory complaints from the self-reports and the informants’ reports, 92.9% of MCI patients and 96% of dementia patients suffered from subjective memory decline, which was significantly different from the value for healthy subjects (odds ratio (OR): 24.3, 95% confidence interval (CI): 15.2–38.7). Comparing MCI patients with dementia patients, the patients themselves did not report more memory complaints, but their informants reported more memory complaints for dementia patients.

### Risk factors

In risk factor evaluation, only head trauma with consciousness change, needing assistance to manage money or medications and depression were significantly different from healthy subjects. There were no differences in BMI, hypertension, diabetes, stroke, and hyperlipidemia. In the relative risk analysis comparing MCI and dementia patients with the healthy group, head trauma with consciousness change (OR: 2.6, 95% CI: 1.1–6.1), needing assistance to manage money or medications (OR: 15.9, 95% CI: 8.0–31.8) and depression (OR: 3.4, 95% CI: 1.9–6.0) showed significant differences.

In these risks of dementia, needing help from others to manage money or medications (OR: 14.4) was outstandingly significant, and was used as an independent condition. Head trauma with consciousness change (OR: 2.6), and depression (OR: 3.4) were significant risk factors; hence we assigned them higher weighting values of 3 according to the odd ratio. Hypertension, diabetes, stroke, and hyperlipidemia were not identified in this study, which may be due to selection bias from the hospitals. However, these factors were identified in previous studies [16–27]. We assigned these comorbidities a weight of 1.

### Risk score (RS)

We calculated the RS according to the demographics, as shown in Table 2, with a total score of 18. The RS was 6.6±1.7 for healthy subjects, 6.9±1.9 for MCI patients, and 8.1±2.3 for dementia patients. Therefore, we defined patients as high-risk when they met one of the following three criteria: 1) subjective memory decline regardless of whether based on the patient’s report or the informants’ report, 2) needing help from others to manage money or medications and 3) total RS greater or equal to 8. According to this definition, 89 healthy subjects (53.6%) were included in the high-risk group. Only eight MCI (3.6%) and four dementia patients (0.9%) were included in the low-risk group, who would have been excluded from further BHT-cog. The sensitivity was 98.1%, and the specificity was 44%.

**Table 2:** The risk score (RS) weight of each risk variable.

	Risk Score (RS)
Age	
50–59	0
60–69	1
70–79	2
80–89	3
≧90	4
Gender	
Male	1
Female	2
BMI (<18)	1
Education (<6 years)	1
Stroke	1
Diabetes	1
Hypertension	1
Hyperlipidemia	1
Head trauma with consciousness change	3
Depression	3
Total	18

### Cognitive test

Table 3 shows the detailed scores for the three groups in the tiny objects cognitive test. The table shows a significant difference in orientation to time, immediate recall of five items, verbal fluency test, delayed recall of five items and CDT in the three groups. Since the mean scores in the verbal fluency test for healthy subjects and dementia patients were 9.5 and 4.7, we set the cutoff scores for the verbal fluency test as 9 and 5, respectively.

**Table 3:** The cognitive test scores for the original BHT in the three groups studied: Healthy, MCI, and dementia subjects.

	Healthy (n = 166)	MCI (n = 225)	Dementia (n = 422)	*p*-value
Orientation to time	3.7±0.7	3.2±1.1*	1.2±1.3*+	0.000
Edu (<6 years)	3.3±1.1	2.3±1.3	0.8±1.1*[Table-fn t003fn002]	0.000
Edu (≧6years)	3.8±0.6	3.4±1.0*[Table-fn t003fn002]	1.4±1.4*+ p	0.000
Immediate recall	3.6±1.2	3.2±1.2*	1.9±1.4*+	0.000
Edu (<6 years)	3.0±1.2	2.5±1.2	1.7±1.4	0.000
Edu (≧6years)	3.7±1.2	3.3±1.1*	2.0±1.4*+ p	0.000
Verbal fluency test	9.5±3.3	8.2±3.2*	4.7±3.2*+	0.000
Edu (<6 years)	6.5±2.6	7.1±2.8	4.2±2.9	0.000
Edu (≧6years)	9.9±3.2	8.5±3.2*	5.0±3.3*+ p	0.000
Delayed recall	3.6±1.4	2.6±1.6*	1.0±1.4*+	0.000
Edu (<6 years)	3.1±1.7	2.6±1.9	1.1±1.4	0.000
Edu (≧6years)	3.7±1.3	2.6±1.6*	1.0±1.3*+ p	0.000
Clock Drawing Test (n)	7.9±2.2 (136)	7.0±2.9 (162)*	5.9±2.9 (196)*+	0.000
Edu (<6 years) (n)	6.8±2.2 (8)	7.6±1.4 (7)	5.2±3.1 (22)	0.099
Edu (≧6years)	8.0±2.2 (128)	6.9±2.9 (155) *	6.0±2.8 (174) *+ p	0.000

Note: Data are presented as mean ± standard deviation. The p-value was obtained from one-way analysis of variance. Abbreviations: MCI: mild cognitive impairment

* p < 0.05 compared with control

+ p < 0.05 comparing MCI with dementia

The CDT was not completed by 39.2% (n = 319) of the participants. Among healthy subjects, 20.5% (n = 30) were unable to complete the CDT. Moreover, among healthy subjects with low education, 60% (12/20) could not complete the test. Therefore, we removed the CDT from the short cognitive screening test.

The final version of the BHT-cog consists of assessments for orientation to time, immediate recall, verbal fluency, and delayed recall. The total scores are 4 for orientation to time, 5 for immediate recall of five items, 2 for the verbal fluency test (<5 were 0; between 5 and 8 were 1; > = 9 were 2), and 5 for delayed recall of five items. The revised total score for BHT-cog is 16.

Table 4 presents the results for the finalized BHT-cog, with CDT excluded, for the three groups. The revised total score for BHT-cog is 16. The healthy subjects scored 12.5±2.6, MCI patients scored 10.3±2.9, and dementia patients scored 4.7±3.3, with mild dementia patients scoring 5.3±3.0. Among the patients with low education, healthy subjects scored 10.4±3.3, MCI patients scored 8.5±3.4, and dementia patients scored 4.1±3.1, with mild dementia patients scoring 4.6±3.0. Among those with a higher level of education (≧6 years), healthy subjects scored 12.7±2.3, MCI patients scored 10.7±2.7, and dementia patients scored 5.0±3.4, with mild dementia patients scoring 5.5±3.0.

**Table 4:** The scores for the finalized BHT-cog in the three groups: healthy, MCI, and dementia subjects.

	Healthy (n = 166)	MCI (n = 225)	Dementia (n = 422)	*p* value
All	12.5±2.6	10.3±2.9*	4.7±3.3*+	0.000
Education (<6 years)	10.4±3.3	8.5±3.4*	4.1±3.1*+	0.000
Education (≧6 years)	12.7±2.3	10.7±2.7*	5.0±3.4*+	0.000

Note: Data are presented as mean ± standard deviation. The p-value was obtained from one-way analysis of variance. Abbreviations: MCI: mild cognitive impairment

* p < 0.05 compared with control

+ p < 0.05 comparing MCI with dementia

### Validity of BHT

**Fig 1:**
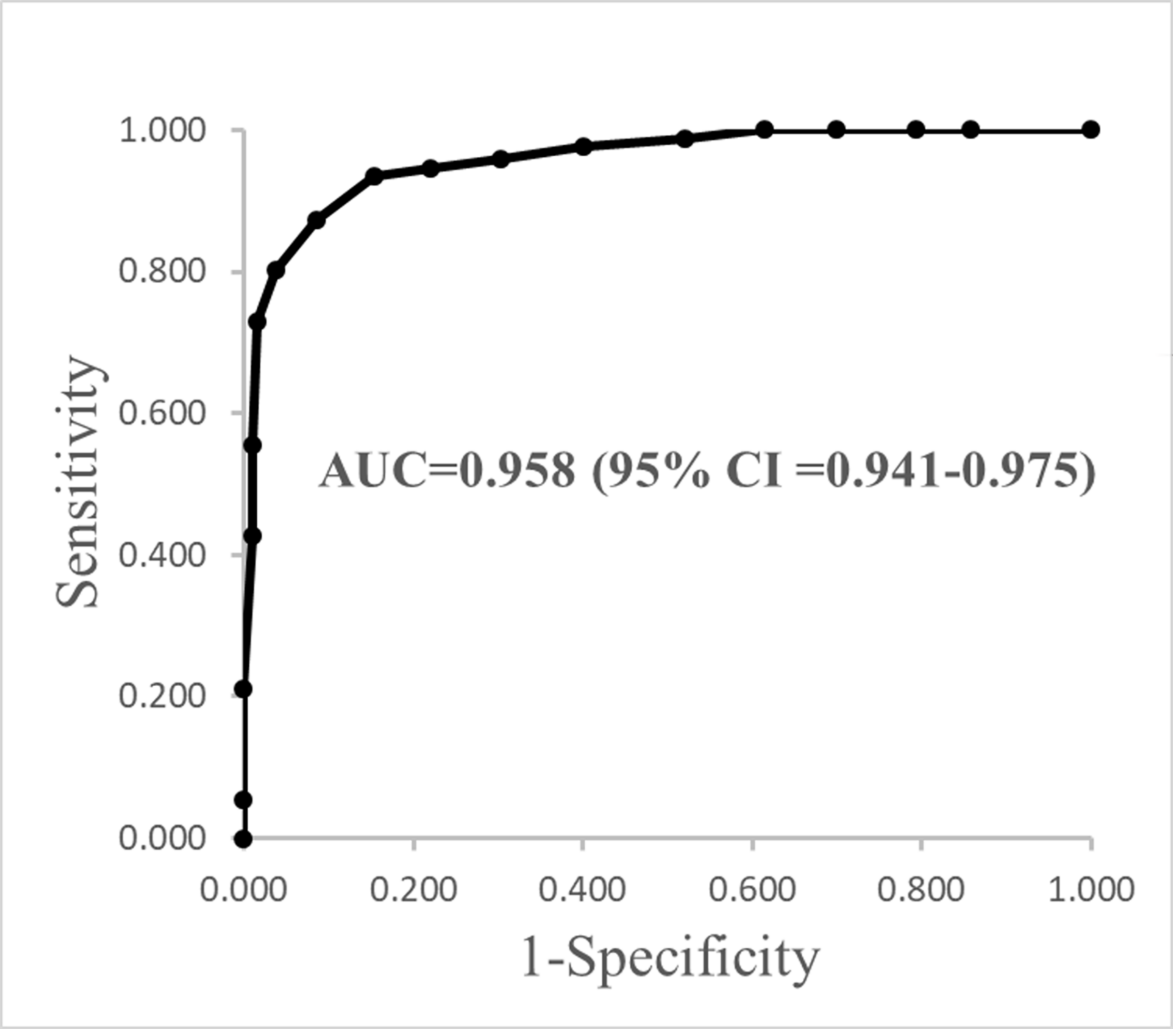
The ROC curve for BHT-cog scores between dementia and healthy subjects. The area under the ROC curve is 0.958 (95% CI = 0.941–0.975). The sensitivity is 91.5%, the specificity is 87.3%, the positive predictive value (PPV) is 94.8%, and the negative predictive value (NPV) is 80.1% while the cutoff value is 10.

We further examined the power of BHT-cog to differentiate demented from healthy subjects using the ROC curve (Fig 1). When the cutoff value was set to 10 (greater or equal to 10 is negative and less than 10 is positive), the area under the ROC curve was 0.958 (95% CI = 0.941–0.975). The sensitivity was 91.5%, the specificity was 87.3%, the positive predictive value (PPV) was 94.8%, and the negative predictive value (NPV) was 80.1%. In the subgroup analysis of the high and low education groups, the optimal cutoff value was also 10. The degree of education did not affect the cutoff value.

The area under the ROC curve between MCI and dementia patients was 0.889 (95% CI = 0.864–0.915). The sensitivity was 91.5%, the specificity was 64.9%, the PPV was 83.0%, and the NPV was 80.2% when the cutoff value was set to 10. In the subgroup analysis of the high and low education groups, the optimal cutoff value was also 10. The degree of education did not affect the cutoff value, but lower NPV and lower specificity were obtained for the low education group (in low education group: sensitivity: 93.2%, specificity: 50%, PPV: 85.8%, NPV: 69.2%; in high education group: sensitivity: 90.1%, specificity: 67.7%, PPV: 82.0%, NPV: 82.1%).

The time requirement for BHT was approximately 4 minutes. The moderately demented patient took longer than normal subjects and severely demented patients.

If we combined the RS and BHT-cog to differentiate normal subjects from those with dementia using a cutoff value of 10, the sensitivity was 90.8%, and the specificity was 92.2% (PPV: 96.7% and NPV: 79.7%).

## Discussion

Early recognition of dementia allows for diagnosis and appropriate treatment, education, psychosocial support, and engagement in shared decision-making regarding life planning, health care, involvement in research, and financial matters. The Alzheimer's Association’s recommendation for operationalizing the detection of cognitive impairment during the Medicare Annual Wellness Visit is to identify high-risk patients to target for cognitive screening[6]. The BHT was developed under this principle, to first identify high-risk patients based on subjective memory complaints and risk factors and then to perform a simple, timely test to detect cognitively impaired patients.

A recent study suggested detecting dementia by case-finding based on subjective memory complaints [37]. However, we only found that 67.8% of dementia patients self-reported memory decline. Alternatively, we found significant differences in subjective memory decline complaints, no matter from subjects or their informants (OR: 24.3), in this study. This difference may be due to the culture. Therefore, for the core feature of dementia, namely memory impairment, we used the responses from subjects and informants as an independent condition.

**Fig 2:**
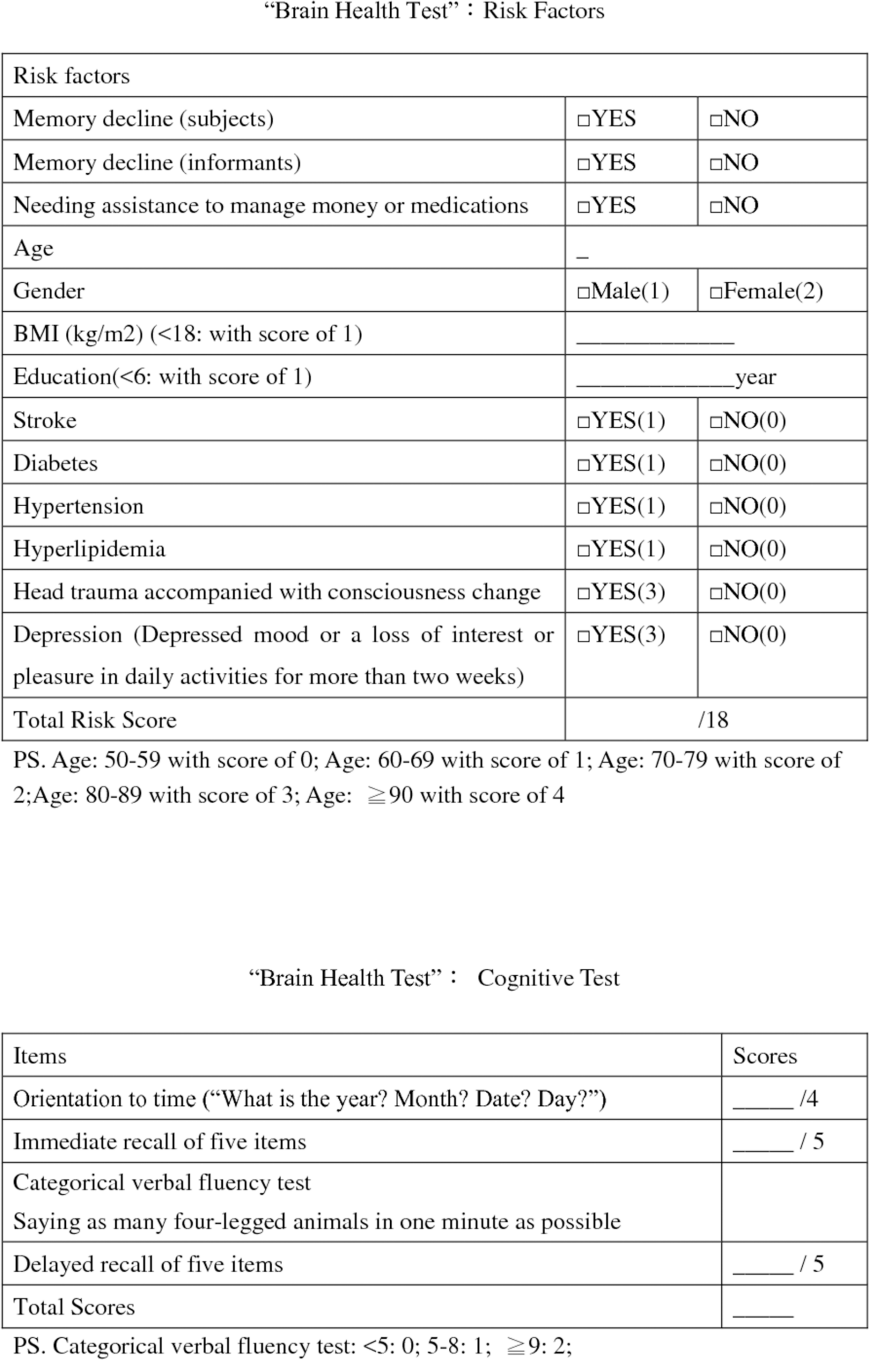
The finalized BHT, English version.

We classified patients with subjective memory decline, regardless of whether the assessment was based on the patient's report or the informants' report, needing help from others to manage money or medications and total RS greater or equal to 8 as the high-risk group. By this definition, only one-half of the healthy subjects were screened, and very few (1.9%) patients with cognitive function impairment were missed. We were able to avoid undesirable adverse psychological effects from screening and adverse effects from false-positive testing. The English version of the revised BHT is attached in Fig 2.

Although AD8 [35], an informant-based screening tool, has been validated in Taiwan with a sensitivity of 97.6% and specificity of 78.1%, there is copyright. Moreover, in Taiwan, there was not a patient-based test, which is more suitable in clinical visiting, because of most of patients coming alone without informants during visiting. We did not adopt MMSE[31, 38] or Montreal Cognitive Assessment[39], that were performed in hospitals, as a mass screening tool in the community to avoid the duplication. Mini-cognitive assessment instrument (Mini-Cog) test[40], that has been validated in Taiwan with a low sensitivity of 53.7% and a high specificity of 95.5%, was not suitable for screening in the community. And last, the major difference between the BHT and the above examinations was the risk evaluation, which was able to avoid undesirable adverse psychological effects from screening and adverse effects from false-positive testing.

Formal assessments of mental status and tests of global cognitive function almost invariably include measures of temporal orientation and episodic memory. Item analyses of the MMSE have demonstrated that among all elements, word recall and temporal orientation have the best properties for detecting dementia [8]. These two elements were included in the BHT-cog. BHT-cog showed a high correlation with MMSE, with a correlation coefficient of 0.821 (R^2^ = 0.675). The time requirement for BHT is only about 4 minutes, which is its greatest advantage for mass screening in the community.

Although a previous study reported that CDT was a suitable screening tool for cognitive impairment, we did not obtain similar results in this study [41, 42]. Possible reasons are differences between urban and rural settings and the difficulties we experienced with introducing CDT to patients.

The cost of treating comorbid conditions in AD patients is $3000 per year higher than that for age-matched patients[43]. From a logical and evidence-based medicine perspective, there is no longer any question that early detection and treatment are both humane and cost-effective for families, patients, physicians, healthcare providers, and payer[44]. Besides the high sensitivity and specificity, the high NPV of BHT is another benefit. This means that if mass opportunistic screenings during community health checks are performed with BHT, community-based non-pharmacologic therapy, such as cognitive occupational therapy[45, 46], reminiscence therapy[47–49], exercise therapy[45, 50], etc., which have been developed to stimulate cognitive abilities, slow cognitive deterioration, reduce problematic behaviors, and improve the quality of life for patients with dementia, could be provided as soon as possible.

This study is a hospital-based study, including both medical centers and community hospitals. These hospitals are located throughout the island, in the northern, middle, southern, and eastern parts of Taiwan. The participants included urban, rural, low education, and high education patients. BHT should therefore be applicable to the elder population in Taiwan with different demographic backgrounds.

There are some limitations to the study. Most of the dementia patients (75.8%) included in this study had AD. BHT cannot be used as a tool for differentiating the different subtypes of dementia, which should be confirmed by dementia specialists. Another weak point is that the ability of BHT to separate healthy subject from those with MCI was only moderate. The area under the ROC curve between the healthy subjects and MCI patients was 0.721 (95% CI = 0.670–0.772). The PPV was 74.7%, and the NPV was 56.8% with a cutoff value of 12.

## Conclusion

There are too few dementia specialists to ensure the correct diagnosis in Taiwan, as with the rest of the world[51]. Therefore, an opportunistic screening tool that can be used for dementia screening in community health checks to assist primary care physicians and public health nurses is urgently needed. The BHT, developed in this study, was designed for the screening of dementia patients in the community by primary care physicians and public health nurses, who do not receive comprehensive cognitive assessment training. The test showed high sensitivity and specificity in differentiating dementia patients from MCI and healthy subjects. Primary care physicians and public health nurses were amenable to all the elements in BHT. Its simplicity makes it a convenient tool for opportunistic screening in community health checks.

## Supporting information

S1 Fig The original BHT questionnaire modified in this study. (PDF)

S1 Data The raw data used in this study. (XLSX)
