# Supplementary material for: Development and validation of a dementia screening tool for primary care in Taiwan: Brain Health Test
Source: PLoS One. 2018 Apr 25;13(4):e0196214. doi: 10.1371/journal.pone.0196214 (PMC5918945; doi:10.1371/journal.pone.0196214)
Supplement: S1 Fig — (PDF) [file pone.0196214.s001.pdf]

Brain Health Test: Risk Factors

Name	______________________	
Birthday	_________________________	
Performed Date	_________________________	
Medical ID	_________________________	
ID	_________________________	
Risk factors		
Memory decline (subjects)	□YES	□NO
Memory decline (informants)	□YES	□NO
Memory impairment (doctors)	□YES	□NO
Age	___	
Gender	□Male	□Female
BMI (kg/m^2)	______________	
Education	______________year	
Diabetes	□YES	□NO
Hypertension	□YES	□NO
Hyperlipidemia	□YES	□NO
Head trauma accompanied by consciousness change	□YES	□NO
Needing assistance to manage money or medications	□YES	□NO

Depression (Depressed mood or a loss of interest or pleasure in daily activities for more than two weeks)	□YES	□NO

TDS “Brain Health Test”： Cognitive Test

Items	Scores
Orientation to time (“What is the year? Month? Date? Day?”)	_____ /4
Immediate recall of five items	_____ / 5
Categorical verbal fluency test<br>Saying as many four-legged animals in one minute as possible	
Delayed recall of five items	_____ / 5
Clock Drawing Test (10:10)	
Total Score	_____
